# PragmaTic, prospEctive, randomized, controlled, double-blind, mulTi-centre, multinational study on the safety and efficacy of a 6% HydroxYethyl Starch (HES) solution versus an electrolyte solution in trauma patients: study protocol for the TETHYS study

**DOI:** 10.1186/s13063-022-06390-x

**Published:** 2022-06-02

**Authors:** Clementina Duran Palma, Musawenkosi Mamba, Johan Geldenhuys, Oluwafolajimi Fadahun, Rolf Rossaint, Kai Zacharowski, Martin Brand, Óscar Díaz-Cambronero, Javier Belda, Martin Westphal, Ute Brauer, Dirk Dormann, Tamara Dehnhardt, Martin Hernandez-Gonzalez, Sonja Schmier, Dianne de Korte, Frank Plani, Wolfgang Buhre

**Affiliations:** 1 Trident Clinical Homestead Medical Center, Kimberley, South Africa; 2 Gama Research Center, Soshanguve, Gauteng South Africa; 3 FCRN Clinical Trials Centre, Vereeniging, South Africa; 4 Gama Research Centre, Leratong Hospital, Johannesburg, South Africa; 5 RWTH University Hospital, Rhineland-Westfalen Technical University, Aachen, Germany; 6 Frankfurt University Hospital, Johannes Goethe University, Frankfurt, Germany; 7 Steve Biko Academic Hospital, Pretoria, South Africa; 8 Universitari i Politècnic La Fe Hospital, University of Valencia, Valencia, Spain; 9 Hospital Clínico Universitario, University of Valencia, Valencia, Spain; 10 Fresenius Kabi Deutschland GmbH, Bad Homburg, Germany; 11 B. Braun Melsungen AG, Melsungen, Germany; 12 Division of Acute and Critical Medicine, Maastricht University Medical Centre, Maastricht, the Netherlands; 13 Department of Anesthesiology and Pain Medicine, Maastricht University Medical Centre, Maastricht, the Netherlands; 14 Chris Hani Baragwanath Hospital, Soweto, South Africa

**Keywords:** Volume therapy, Colloids, Hydroxyethyl starch, HES, Trauma, Blood loss, Acute kidney injury

## Abstract

**Background:**

Trauma may be associated with significant to life-threatening blood loss, which in turn may increase the risk of complications and death, particularly in the absence of adequate treatment. Hydroxyethyl starch (HES) solutions are used for volume therapy to treat hypovolemia due to acute blood loss to maintain or re-establish hemodynamic stability with the ultimate goal to avoid organ hypoperfusion and cardiovascular collapse. The current study compares a 6% HES 130 solution (Volulyte 6%) versus an electrolyte solution (Ionolyte) for volume replacement therapy in adult patients with traumatic injuries, as requested by the European Medicines Agency to gain more insights into the safety and efficacy of HES in the setting of trauma care.

**Methods:**

TETHYS is a pragmatic, prospective, randomized, controlled, double-blind, multicenter, multinational trial performed in two parallel groups. Eligible consenting adults ≥ 18 years, with an estimated blood loss of ≥ 500 ml, and in whom initial surgery is deemed necessary within 24 h after blunt or penetrating trauma, will be randomized to receive intravenous treatment at an individualized dose with either a 6% HES 130, or an electrolyte solution, for a maximum of 24 h or until reaching the maximum daily dose of 30 ml/kg body weight, whatever occurs first. Sample size is estimated as 175 patients per group, 350 patients total (*α* = 0.025 one-tailed, power 1–*β* = 0.8). Composite primary endpoint evaluated in an exploratory manner will be 90-day mortality and 90-day renal failure, defined as AKIN stage ≥ 2, RIFLE injury/failure stage, or use of renal replacement therapy (RRT) during the first 3 months. Secondary efficacy and safety endpoints are fluid administration and balance, changes in vital signs and hemodynamic status, changes in laboratory parameters including renal function, coagulation, and inflammation biomarkers, incidence of adverse events during treatment period, hospital, and intensive care unit (ICU) length of stay, fitness for ICU or hospital discharge, and duration of mechanical ventilation and/or RRT.

**Discussion:**

This pragmatic study will increase the evidence on safety and efficacy of 6% HES 130 for treatment of hypovolemia secondary to acute blood loss in trauma patients.

**Trial registration:**

Registered in EudraCT, No.: 2016-002176-27 (21 April 2017) and ClinicalTrials.gov, ID: NCT03338218 (09 November 2017).

## Background

Traumatic injuries are a relevant cause of morbidity and mortality worldwide. Regardless of its nature, trauma represents a serious clinical condition, often associated with loss of vascular integrity and bleeding [1, 2]. A decrease of circulating blood volume (hypovolemia) may result in hemodynamic instability, decreased tissue perfusion, cellular hypoxia, organ damage, and finally death [3]. The aim of volume replacement therapy is to counteract hypovolemia and maintain hemodynamics, increasing oxygen delivery to tissues and restoring/preserving vital functions by matching tissue oxygen demand with supply [4, 5].

Crystalloid solutions (composed of water and electrolytes) and colloid solutions (containing macromolecules such as hydroxyethyl starch (HES), gelatin, or albumin) are routinely used for volume therapy, particularly in trauma settings with acute blood loss [6, 7]. While crystalloid solutions diffuse easily into the interstitial space, colloid solutions contain macromolecules that are less likely to pass semi-permeable biological membranes. From a physiologic point of view, this oncotic effect translates into pronounced hemodynamic effects at lower volumes, with decreased likelihood of tissue edema and complications resulting from fluid overload as compared to crystalloids [6, 8–12].

Since three investigator-initiated studies [13–15], however, reported an increased risk of renal impairment and mortality in critically ill patients, the European Medicine Agency (EMA) requested all marketing authorization holders (MAH) of HES-containing medicinal products in the European Union to develop a clinical trial strategy to provide long-term safety data of HES in the perioperative setting and trauma [16]. Accordingly, Fresenius Kabi and B. Braun Melsungen decided to perform two clinical studies to address the safety and efficacy of the use of HES: (1) the Prospective, randomized, controlled, double-blind, multi-center, multinational study on the safety and efficacy of 6% Hydroxyethyl starch (HES) sOlution versus an Electrolyte solutioN In patients undergoing eleCtive abdominal Surgery (PHOENICS) trial [17] and (2) this supportive PragmaTic, prospEctive, randomized, controlled, double-blind, mulTi-centre, multinational study on the safety and efficacy of a 6% HydroxYethyl Starch (HES) solution versus an electrolyte solution in trauma patients (TETHYS).

### Study objective

The primary objective of this study is to evaluate the safety of a 6% HES 130 solution compared to an electrolyte solution for infusion in patients with trauma, using an exploratory non-inferiority design. Secondary objectives are to further assess efficacy and safety, as determined by renal function, blood coagulation, inflammation, hemodynamics, total administered investigational product (IP) volume over the study time, fluid balance, concomitant medication, need of blood products, (serious) adverse events, and additional outcome parameters.

## Methods/design

### Trial design

TETHYS is a pragmatic, prospective, randomized, controlled, double-blind, multi-center, multinational study, performed in two parallel groups of patients admitted to general hospitals, and allocated in a 1:1 fashion to receive either a 6% HES 130 solution (Volulyte® 6%, Fresenius Kabi Deutschland GmbH, Bad Homburg, Germany; HES group) or an electrolyte solution (Ionolyte®, Fresenius Kabi Deutschland GmbH, Bad Homburg, Germany; control group). The study is conducted as a phase IV study in most European countries of conduct; in Serbia, Czech Republic, and South Africa, it is a phase III study. Independent ethical committee (IEC) approval to the corresponding protocol versions has been already granted in most of the participants’ countries. The corresponding study protocols are described in this publication in accordance with the SPIRIT Guidelines [18].

### Participants

The study is conducted in a population of adult (≥18 years of age) patients, both genders, presenting with blunt or penetrating trauma, an estimated blood loss of ≥ 500 ml, an indication of surgery within 24 h after trauma, and no signs of intracranial or cerebral hemorrhage. Eligible patients must consent to participate in the trial, either personally, or following a deferred written informed consent, or as locally required. Patients must not have received ≥ 15 ml/kg body weight of colloids between trauma injury and hospital admission. Females of childbearing potential must test negative on a standard pregnancy test to participate in the study. Patients are also excluded from the trial if meeting any of the following criteria: known or suspected hypersensitivity to any of the investigational product or its excipients, body weight of ≥ 140 kg, expectancy of death within 24 h after traumatic injury, sepsis, burns, renal and impairment at time of admission (defined as AKIN stage ≥ 1, or chronic kidney failure). The same is true for patients receiving acute or chronic renal replacement therapy (RRT), critically ill patients (typically admitted to the intensive care unit, ICU), or patients suffering from hyperhydration, pulmonary edema, dehydration, hyperkalemia, severe hypernatremia, severe hyperchloremia, severely impaired hepatic function, congestive heart failure, severe coagulopathy, organ transplant patients, and metabolic alkalosis. Trial participation is also excluded for patients with simultaneous participation in another interventional clinical trial for either drugs or medical devices.

In line with the scientific advice obtained from the EMA Scientific Advice Working Party, it is aimed to recruit as many patients as possible (up to 350 patients) within the recruitment period of the concurrent perioperative study PHOENICS [17]. A list of the participating sites in TETHYS can be found at Clinicaltrials.gov (NCT03338218).

### Investigational products (IPs)

The investigational test product Volulyte® 6% is a clear to slightly opalescent, colorless 6% HES 130/0.4 solution in an isotonic, balanced electrolyte solution. The investigational reference product Ionolyte® is a clear and colorless, aqueous, balanced electrolyte solution. Since Ionolyte® has the identical electrolyte composition as Volulyte® 6%, it is the most suitable comparator. Both products are solutions for infusion provided in 500 ml polyolefin bag (Freeflex®) with overwrap.

### Study phases and interventions

A detailed schematic of all study procedures is shown in Table 1.

**Table 1:** TETHYS study flow diagram

	Time					
	Screening: at hospital admission	T0 (baseline): emergency room until IP treatment start	T1: First 24 h after IP treatment start^b^	T2: post-traumatic day 1^b^-3 (morning)	T3: post-traumatic day 4-7 (morning)	T4^c^: day 90 after randomization
**Procedure**						
Inclusion/exclusion criteria	X					
Randomization		X				
Demographic data and medical history	X					
Anamnesis and concomitant diseases (only ongoing and relevant resolved)	X					
Date and time of hospital admission	X					
Blunt/penetrating trauma	X					
Injury characteristics	X					
Fluid input (colloids, crystalloids) after trauma injury and until hospital admission	X					
Surgery due to traumatic injury						
• Type of surgery • Date • Time of skin incision/time of skin suture			X	X	X	
SCr [mg/dl] SCr-based eGFR [ml/min] Cystatin-C [mg/dl] Cystatin-C based eGFR [ml/min] Cystatin-C-based mean eGFR [ml/min] (calculated from the highest cystatin-C level during days 1–3) AKIN score (calculated) Highest AKIN stage reached on each day (during the first week) (calculated) RIFLE score (calculated) Urine output (if available)		X	X^a^	X	X^d^	
C-reactive protein [mg/L]		X	X^a^	X		
Platelet count [μ/L] INR aPTT [s]		X	X^a^	X		
pCO_2_ [mmHg] pO_2_ [mmHg] HCO_3_^-^ [mmol/l] SaO_2_ [%] pH Base excess [mEq/l] Hb [g/dl] Hct [%] Lactate [mmol/l]		X	X^a^	X		
ScvO_2_ [%] (if available)		X	X^a^	X		
Na^+^ [mmol/l] K^+^ [mmol/l] Ca^2+^ [mmol/l] Cl^-^ [mmol/l]		X	X^a^	X		
Administered IP volume [19]		X	X			
Fluid input [19] (incl. every i.v. medication, applied blood products) Fluid output [19] (incl. drainage, urine output, estimated blood loss)		X	X	X	X	
Temperature [°C]		X	X	X	X	
MAP [mmHg] (calculated) HR [beats/min] SAP [mmHg] DAP [mmHg] CVP [mmHg] (if available)		X	X^a^	X	X	
Hemodynamics as required to determine volume responsiveness (one variable if applicable)						
• MAP [mmHg] • SV [19] • SVV [%] • PPV [%] • SVI [ml/min^2^]		During duration of IP administration to assess volume responsiveness				
Mechanical ventilation	X	X	X	X	X	
Use of RRT			X	X	X	X
Antibiotics Contrast agents Diuretics		X	X	X	X	
Crystalloid (including basal infusion)/albumin						
• Administered drug • Volume		X	X	X	X	
Vasoactive/inotropic drugs						
• Administered drug • Dosage/volume		X	X	X	X	
Fibrinogen/PCC/factor XIII						
• Administered drug • Dosage/volume		X	X	X	X	
Applied blood products [19]						
• Administered drug • Dosage/volume		X	X	X	X	
(Serious) adverse events		continuously				
Date and time of hospital discharge					At hospital discharge	
Fulfilment of fit for discharge criteria from hospital					Daily until fulfilment	X
Date of ICU admission		At ICU admission				
Date and time of discharge from ICU				At ICU discharge		
Fulfilment of fit for ICU discharge criteria				Daily until fulfilment		X
Mortality (in-hospital/out of hospital)				X	X	X
Study termination		At termination				

^a^At least every 6 h

^b^In case assessment of time points T1 and T2 (day 1) are within a timespan of max. 2 h only one assessment per variable has to be done to minimize intervention for the patient, otherwise deemed clinically required

^c^A lag time of ± 14 days for the conduct of this follow-up contact is accepted, to account difficulties in obtaining data due to potential causes for delay (e.g., mail delay, or patient´s inability to travel)

^d^except cystatin-C

#### Enrolment (screening, randomization, and baseline)

Patients are screened at hospital admission to verify in- and exclusion criteria, demographic data, medical history, and pregnancy test in women of childbearing potential. Once screened, and after voluntary or deferred informed consent, patients are randomly allocated in a 1:1 fashion into two equal groups (Volulyte® or Ionolyte®) to receive an individualized dosing of IP. Both the investigator(s) and medical staff of the study site and the patients participating in the study are unaware to which treatment arm the patient is allocated.

#### Treatment phase

**Fig. 1:**
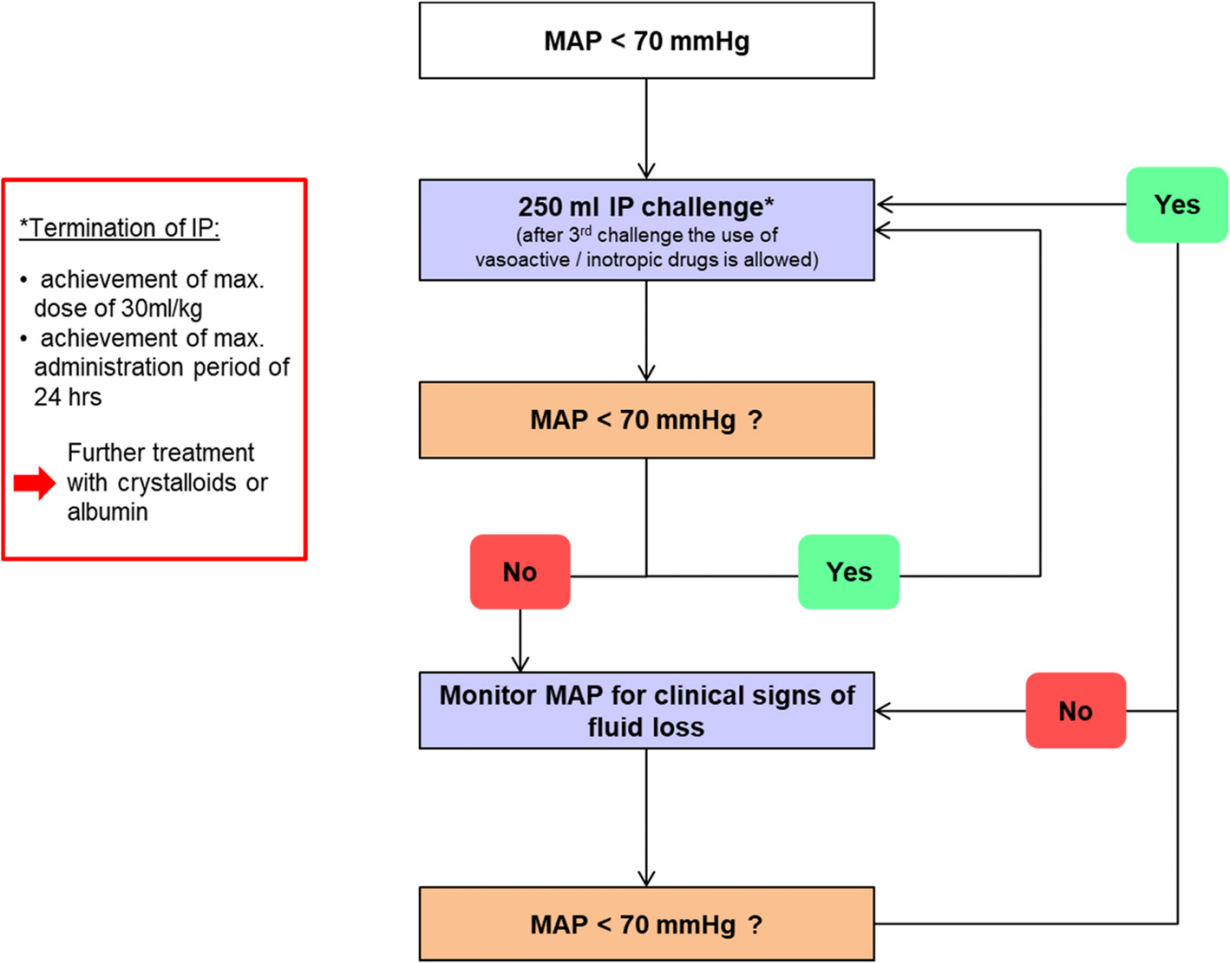
Mean arterial pressure optimization protocol to guide perioperative volume losses. MAP, mean arterial pressure; IP, investigational product (Volulyte or Ionolyte)

**Fig. 2:**
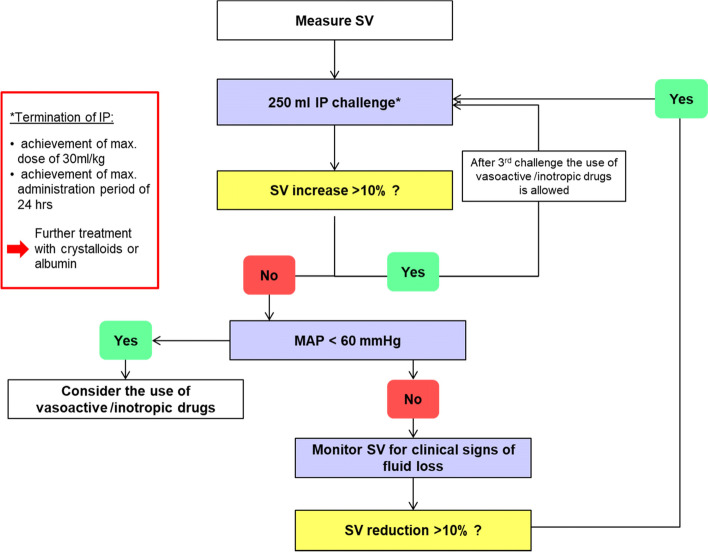
Stroke volume optimization protocol to guide perioperative volume losses (adapted from Kuper, Martin, et al. “Intraoperative fluid management guided by esophageal Doppler monitoring.” Bmj 342 (2011)). SV, stroke volume; MAP, mean arterial pressure; IP, investigational product (Volulyte or Ionolyte)

**Fig. 3:**
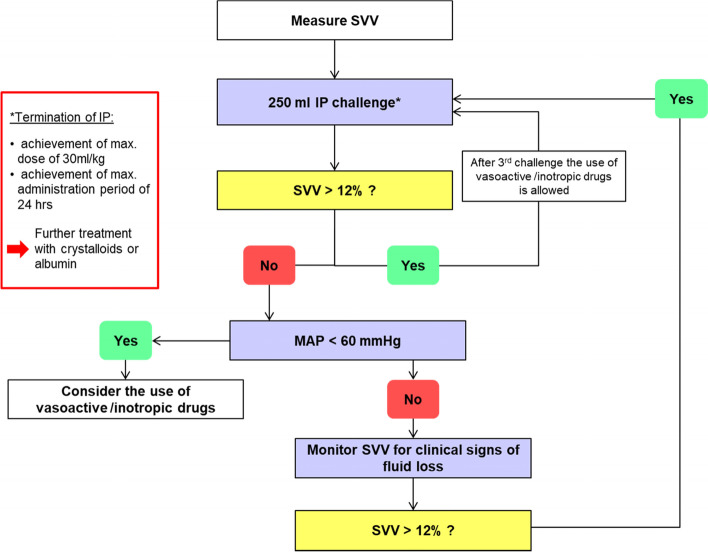
Stroke volume variation optimization protocol to guide perioperative volume losses. VV, stroke volume variation; MAP, mean arterial pressure; IP, investigational product (Volulyte or Ionolyte)

During the treatment phase, IP will be administered intravenously according to the patient volume needs, preferably guided by an algorithm based on either mean arterial pressure or dynamic circulatory parameters, whichever preferred by the treating physician (Algorithms are shown in Figs. 1, 2, and 3). The choice of the hemodynamic stabilization algorithm and the definition of volume responsiveness relies with the local investigator at each site at the beginning of the study and has to be followed for both groups during the whole study period within the study site; in case a contraindication to the use of any dynamic monitoring device arises, the investigator is allowed to switch to a valid alternative (e.g., mean arterial pressure, MAP), which must be properly documented.

Treatment with IP is provided until a maximum of 24 h after start of its administration, or until reaching a maximum daily dose of 30 mL/kg, whatever occurs first. In case the patient is still hypotensive during IP administration, vasoactive/inotropic drugs can be administered, if considered necessary. As HES preparations for volume replacement rarely cause allergic reactions of varying severity, the first 10-20 ml of IP are infused slowly. In case of an allergic reaction, the infusion is stopped immediately, and appropriate treatment is to be initiated.

In case the patient is not hemodynamically stabilized after having received the maximum allowed daily IP dosage of 30 ml/kg or 24 h after IP treatment start (whatever occurs first), only crystalloid solutions, or albumin are to be administered. The choice of the solution is at the discretion of the treating physician and shall be documented in the electronic case report form (eCRF), including administered volume. If needed, blood products can be given throughout the study period and should be transfused in accordance with current ESAIC guideline [20], recommending a target hemoglobin concentration of 7-9 g/dl during active bleeding, or, in South African sites, according to the current version of the Clinical Guidelines for the Use of Blood Products in South Africa by the South African National Blood Transfusion Service [21], which recommend administration of red blood cell component (RBC) if the acute blood loss is greater than 30% of blood volume, until reaching a preoperatory target hemoglobin concentration between 7 and 10 g/dl; RBC transfusions above 10 g/dL are not recommended.

#### Study assessments and follow-up

Study data are collected at different time points during the study, from baseline (after hospital admission, at emergency room until IP treatment start), up to 7 days after trauma, followed by a period of 90 ± 14 days after randomization. A detailed schematic showing the time points for study assessments and procedures, including follow-up, is presented in Table 1.

### Concomitant medication

#### Allowed concomitant medication

The following medications are allowed, as long as they are provided solely via a separate infusion system, independently from the IP infusion system:

- Vasoactive/inotropic treatment, starting earliest after third IP volume challenge or if clinically deemed necessary
- Medication that is clinically required, except other volume replacement therapy (either colloids or crystalloids) during IP treatment period, e.g., antibiotics or pain medication
- Crystalloid solutions or albumin, if clinically required, after treatment phase, i.e., after achieving the maximum daily dose of 30 ml/kg IP or maximum IP treatment period of 24 h, whatever occurs first
- If concomitant blood products are necessary, these should only be given according to the most current version of either the ESAIC guideline on the management of severe perioperative bleeding [20], or the Clinical Guidelines for the Use of Blood Products in South Africa by the South African National Blood Transfusion Service [21], wherever applicable

#### Not allowed concomitant medication

- Any colloid (i.e., gelatin solutions, albumin, dextran, other HES solutions) during IP-treatment phase
- Synthetic colloids (i.e., gelatin solutions, dextran, and other HES solutions) after the IP-treatment phase until hospital discharge
- Intravenous crystalloid solutions during IP treatment phase besides basal infusion

### Outcomes

#### Primary outcome

The primary outcome is a composite of 90-day mortality and 90-day renal failure, defined by biomarker increase according to AKIN [22] stage ≥ 2, RIFLE [23] injury or failure stage, or need for RRT including hemodialysis, peritoneal dialysis, hemofiltration, and renal transplantation at any time during the first 3 months after treatment with IP.

#### Secondary outcomes

The assessment of secondary variables (listed in Table 2) will provide further information on safety and efficacy of both IPs. Date and time of assessment are documented for all variables.

**Table 2:** Secondary variables

Safety parameters	Efficacy parameters	Other variables
**Renal function** • Serum creatinine and method of determination (colorimetric or enzymatic) • Cystatin-C • Serum creatinine-based eGFR • Cystatin-C-based eGFR^a^ • Cystatin-C-based mean eGFR (calculated from the highest cystatin-C level during day 1-3)^a^ • AKIN score^b^ • RIFLE stage^b^ • Urine output (if available) **Coagulation** • Platelet count • International normalized ratio • Activated partial thromboplastin time **Inflammation** • C-reactive protein **Adverse events** • (Serious) adverse events/reactions **Outcome** • Length of stay (LOS): □ LOS in the hospital □ LOS in the intensive care unit^c^ □ Fulfilment of fit for discharge from ICU/hospital^d^ • Hours on mechanical ventilation • In-hospital/out of hospital mortality (incl. cause) • Days on renal replacement therapy	**Fluid administration** • Administration of IP volume **Fluid balance** • Fluid input and output **Hemodynamics/vital signs** • Heart rate • Mean arterial pressure (MAP) • Systolic arterial blood pressure • Diastolic arterial blood pressure • Central venous pressure^c^ *at least one of the following parameters (volume algorithm):* • Stroke volume (SV) • Stroke volume variation (SVV) • Stroke volume index (SVI) • Pulse pressure variation (PPV) • Mean arterial blood pressure (MAP) **Laboratory data** • Arterial (preferred) blood gas analysis □ Partial pressure of carbon dioxide □ Partial pressure of oxygen □ Bicarbonate □ Arterial oxygen saturation □ pH □ Base excess □ Lactate □ Hemoglobin □ Hematocrit Central venous oxygen saturation^c^ • Serum electrolytes □ Sodium □ Potassium □ Calcium □ Chloride	**Demographic data and medical history** • Age • Gender • Height • Weight • Ethnicity • Anamnesis and concomitant diseases (only ongoing and relevant resolved) • Fluid input from trauma injury until hospital admission **Trauma-related data** • Blunt/penetrating trauma • Injury characteristics □ Injury Severity Score □ Glasgow Coma Scale Surgery due to traumatic injury □ Type of surgery **Concomitant medication** • Vasoactive/inotropic drugs • Amount of transfused blood products [19] including specification (if available) • Coagulation factors (i.e., fibrinogen/PCC/factor XIII) • Antibiotic therapy • Contrast agents • Diuretics • Crystalloid solutions/albumin (incl. basal infusion)

^a^Calculated from highest cystatin-C level on days 1–3, or hospital discharge (whatever occurs first)

^b^According to Bagshaw et al. [24]. Missing baseline creatinine levels will be estimated according to the MDRD equation [22]

^c^If applicable/if available

^d^As defined by Marshall et al. [25]

#### Sample size

Only descriptive statistics and explorative analyses will be performed, since this study is designed as pragmatic, and supportive to the PHOENICS [17]. Nonetheless, a power analysis was performed.

The composite endpoint of 90-day mortality and 90-day renal failure serves as primary variable. The rate of events of interest is unknown, therefore we defined 50% as a statistically unfavorable condition. Using a non-inferiority margin of *δ* = 15%, both the null hypothesis (*H*_0_, *E*_*T*_ ≥ *E*_*S*_ + 15%) and the alternative hypothesis (*H*_*A*_, *E*_*T*_ < *E*_*S*_ + 15%), where *E*_*T*_ and *E*_*S*_ denote the event rate for the primary endpoint upon Volulyte® 6% and Ionolyte, respectively, will need to be tested on the level of *α* = 0.025 (one-tailed). Therefore, and based on these assumptions, the sample size estimation results in 175 patients per group, 350 patients in total (*α* = 0.025 one-tailed, power 1–*β* = 0.8; SAS PROC POWER). This is considered as the maximum sample size. Power calculations were performed for different sample sizes, up to a power 1–*β* = 0.6, for which a sample size of 109 patients per group, i.e., 218 patients in total, was therefore determined as the minimum acceptable to provide supportive results. The non-inferiority margin of 15% was chosen based on feasibility aspects, in alignment with the competent regulatory authorities, and, particularly, considering the timelines they set for the duration of two concurrent trials in different populations: this trial, and the concurrent PHOENICS [17].

### Randomization, blinding, and unblinding

Assignment to study treatment is randomized in a 1:1 ratio, stratified by site. An Interactive Response Technology System (IRTS) is used for randomization of patients and IP supply, with the order of assignments chosen at random (random permuted block sizes, e.g., 4, 6, or 8).

Investigators and medical staff as well as study participants are blinded to study treatment. Emergency unblinding will only be done via the IRTS by an investigator and/or dedicated authorized personnel (e.g., Pharmacovigilance Department of the Sponsor).

### Statistical methods

All programming of tables, figures, listings, and statistical analyses will be performed using SASÒ version 9.4 or higher. The planned statistics will be done in accordance with guideline ICH E923. A Statistical Analysis Plan (SAP) will be written and finalized prior to unblinding of the study. The primary analysis will be based on the Full Analysis Set (FAS), which closely follows the intention-to-treat principle and includes patients who received study treatment. Additionally, the primary analysis will be restricted to the Per-Protocol Set (PPS) that focuses on patients who are more compliant with the protocol. Prior to database lock, a Blind Data Review Meeting will be held to allow a review of the clinical study data and decide on the final allocation of patients to the analysis sets. If required, consequences for the statistical analysis will be discussed and documented.

The primary combined endpoint of this pragmatic study will be analyzed exploratively and inferences for the trauma population will be drawn in conjunction with the results of the peri-operative study PHOENICS [17]. This primary endpoint will be tested using a non-inferiority margin of *δ* = 15% and following hypotheses: *H*_0_ (null hypothesis, *E*_*T*_ ≥ *E*_*S*_ + 15%) and *H*_*A*_ (alternative hypothesis, *E*_*T*_ < *E*_*S*_ + 15%), where *E*_*T*_ and *E*_*S*_ denote the event rate for the primary endpoint upon Volulyte® 6% and Ionolyte®, respectively. Non-inferiority will be tested with a significance level of *α* = 0.025, one-tailed. Results of explorative analyses will be presented by *p*-values and two-sided 95% confidence intervals to quantify any treatment differences and to demonstrate non-inferiority of Volulyte® 6% vs. Ionolyte® with respect to the primary endpoint.

Secondary outcomes will be compared by means of descriptive statistics and appropriate statistical tests (time to event analyses (Kaplan-Meier Plots, Cox regression), analysis of covariance, logistic regression, Mann-Whitney *U* test, *χ*^2^ test).

Corresponding *p*-values are to be regarded as exploratory; hence, no adjustments for multiple testing will be made, i.e., with an error of the 1st kind *α* = 0.05.

Further analyses, including subgroups, will be specified in a later SAP.

### Trial ethics and governance

This clinical study is being conducted in accordance with the Declaration of Helsinki and in compliance with the study protocol, good clinical practice (GCP), designated Standard Operating Procedures, and with local laws and regulations relevant in the country of conduct. Study protocol was approved by the respective competent authorities and independent ethic committees involved. The study is registered at the European clinical trial database EudraCT database, No.: 2016-002176-27 and in ClinicalTrials.gov, ID: NCT03338218.

Written informed consent must be obtained from all patients before entering the study, in compliance with regulations mentioned above. The investigator explains the nature, purpose, and risks of the study and provides the patient with a copy of the patient information sheet. The patient is given sufficient time to consider the study’s implications before deciding whether to participate, and is free to withdraw from the study at any given time, without specifically stating any reason. In some participating countries, local regulations allow the patient surrogates or family members to provide consent for participating in the trial on behalf of the patient; this procedure is followed whenever applicable.

Protocol amendments will be submitted to the concerned IECs and competent authorities, in line with pertinent regulatory requirements. Any protocol amendment(s) directly affecting patient participation in the study will be reflected in the informed consent form, and the patients will be asked to re-consent accordingly, unless all study procedures had already been completed in this patient prior to amendment approval.

All patient data obtained in the context of this clinical trial are subject to applicable data protection legislation requirements. The storage of data for statistical assessment shall likewise be performed only under the patient’s study identification number. Only the investigator has the means to identify a patient’s name/other personal details via the study identification number.

Withdrawal of individual patients from treatment or from the study respectively could be decided by the investigator, e.g., in case of the occurrence of an adverse event, in case of a protocol deviation (e.g., dosing regimen, failure to comply with protocol), or according to the judgment of the treating physician/investigator, depending on their individual risk assessment. Similarly, the patient can request to be withdrawn on their own will, in compliance with ICH/GCP principles.

In case a patient does not attend any scheduled visit (i.e., lost to follow-up), reasonable effort should be made to contact this patient in order to complete assessments and/or to evaluate reason for non-appearance. Similarly, if the patient is withdrawn for any reason, all efforts have to be taken to gather safety information related to the study, in line with local regulations.

A Data Safety Monitoring Board, consisting of two clinicians (one of them appointed as chair) and a biometrician not involved in study conduct, are monitoring the progress of this study with focus on safety and, if needed, efficacy data. In addition, an independent audit at any study site may take place at any time during or after the study.

All adverse events and/or adverse drug reactions are to be collected in the corresponding case report forms; authorities are to be notified in a timely manner and in line with applicable pharmacovigilance regulations.

## Discussion

This study aims to provide data regarding safety and efficacy of 6% HES 130/0.4 solutions in hypovolemic trauma patients with acute blood loss, following current therapeutic recommendations for the use of products containing HES 130, respecting approved dosing recommendations and contraindications.

The composite primary exploratory endpoint of this study is 90-day mortality and 90-day renal failure. The latter will be defined not only by AKIN and RIFLE criteria, but also by the use of RRT or renal transplantation. Additionally, the efficacy of HES therapy will be measured in terms of dosing requirements to achieve hemodynamic goals. Coagulopathy, transfusion, and/or additional therapeutic requirements will be assessed to determine safety. Further meaningful endpoints (e.g., inflammation, hemodynamic variables, serum electrolytes, serum lactate levels, central venous oxygen saturation, ICU, and hospital length of stay) will be compared among groups to further obtain safety and efficacy data.

Recent meta-analyses of studies assessing the risk of renal impairment after HES administration, including those performed in trauma patients, have not shown significant differences in postoperative mortality and kidney disfunction [26, 27], but a favorable trend towards a reduced hospital length of stay and lower vasopressors requirements for hemodynamic stability during resuscitation in patients treated with HES. The Fluids In Resuscitation of Severe Trauma (FIRST) trial [28] compared the effect of HES 130/0.4 versus normal saline solution in blunt and penetrating trauma and found comparable efficacy in achieving hemodynamic goals, with better lactate clearance, lower kidney injury rates, and lower alteration in coagulation parameters in patients with penetrating trauma treated with HES 130/0.4, compared to those treated with normal saline. However, these effects were not observed in patients with blunt trauma.

Analyses of safety outcomes in trauma subpopulations from studies in surgical and/or critically ill patients have not been decisive, especially due to small patient numbers, higher median age, and a number of pre-existing comorbidities that increase the risk of complications, particularly renal impairment [29–32]. Therefore, and to fill this evidence gap, this pragmatic, exploratory study assesses the safety and efficacy of HES 130 administration in trauma patients requiring volume therapy due to acute blood loss [33].

## Trial status

This clinical study is currently in the recruitment phase.

## Data Availability

A decision about making the data public available has not been taken yet.

## Abbreviations

AKIN: Acute Kidney Injury Network
eCRF: Electronic case report form
CT.gov: Clinical Trials Database (ClinicalTrials.gov)
CTN: Clinical Trials Network
CRO: Contract Research Organization
CVP: Central venous pressure
EMA: European Medicines Agency
ESAIC: European Society of Anesthesia and Intensive Care
EudraCT: European Union Drug Regulating Authorities’ Clinical Trials database
GCP: Good Clinical Practices
HES: Hydroxyethyl starch
ICH: International Committee for Harmonization
ICU: Intensive care unit
IEC: Independent ethical committee
IP: Investigational product
IRTS: Interactive Response Technology System
MAP: Mean arterial pressure
PHOENICS: Acronym of the Prospective, randomized, controlled, double-blind, multi-center, multinational study on the safety and efficacy of 6% Hydroxyethyl starch (HES) sOlution versus an Electrolyte solutioN In patients undergoing eleCtive abdominal Surgery
PPV: Pulse pressure variation
RIFLE: Risk, Injury, Failure, Loss, End-stage classification of acute kidney injury
RRT: Renal replacement therapy
SANBS: South African National Blood Service
SAP: Statistical Analysis Plan
SVI: Stroke volume index
SVV: Stroke volume variation
TETHYS: Acronym for the PragmaTic, prospEctive, randomized, controlled, double-blind, mulTi-centre, multinational study on the safety and efficacy of a 6% HydroxYethyl Starch (HES) solution versus an electrolyte solution in trauma patients
